# Interspecific cytogenetic relationships in three *Acestrohynchus* species (Acestrohynchinae, Characiformes) reveal the existence of possible cryptic species

**DOI:** 10.3897/CompCytogen.v14i1.33483

**Published:** 2020-01-15

**Authors:** Alber Sousa Campos, Ramon Marin Favarato, Eliana Feldberg

**Affiliations:** 1 Programa de Pós-Graduação em Genética, Conservação e Biologia Evolutiva (PPG GCBEv). Coordenação de Biodiversidade, Instituto Nacional de Pesquisas da Amazônia, , Av. André Araújo, 2936, Petrópolis, Manaus, Amazonas, Brazil Instituto Nacional de Pesquisas da Amazônia Manaus Brazil

**Keywords:** Repetitive DNA, fish cytotaxonomy, FISH, ITS

## Abstract

The karyotypes and chromosomal characteristics of three *Acestrorhynchus* Eigenmann et Kennedy, 1903 species were examined using conventional and molecular protocols. These species had invariably a diploid chromosome number 2n = 50. *Acestrorhynchus falcatus* (Block, 1794) and *Acestrorhynchus falcirostris* (Cuvier, 1819) had the karyotype composed of 16 metacentric (m) + 28 submetacentric (sm) + 6 subtelocentric (st) chromosomes while *Acestrorhynchus microlepis* (Schomburgk, 1841) had the karyotype composed of 14m+30sm+6st elements. In this species, differences of the conventional and molecular markers between the populations of Catalão Lake (AM) and of Apeu Stream (PA) were found. Thus the individuals of Pará (Apeu) were named Acestrorhynchus prope microlepis. The distribution of the constitutive heterochromatin blocks was species-specific, with C-positive bands in the centromeric and telomeric regions of a number of different chromosomes, as well as in interstitial sites and completely heterochromatic arms. The phenotypes of nucleolus organizer region (NOR) were simple, i. e. in a terminal position on the *p* arm of pair No. 23 except in *A. microlepis*, in which it was located on the *q* arm. Fluorescence *in situ* hybridization (FISH) revealed 18S rDNA sites on one chromosome pair in karyotype of *A. falcirostris* and A. prope microlepis (pair No. 23) and three pairs (Nos. 12, 23, 24) in *A. falcatus* and (Nos. 8, 23, 24) in *A. microlepis*; 5S rDNA sites were detected in one chromosome pair in all three species. The mapping of the telomeric sequences revealed terminal sequences in all the chromosomes, as well as the presence of interstitial telomeric sequences (ITSs) in a number of chromosome pairs. The cytogenetic data recorded in the present study indicate that A. prope microlepis may be an unnamed species.

## Introduction

The family Acestrorhynchidae is a group of South American fishes, for which contradictory views on its phylogenetic position within Characiformes are debated. Based on the analysis of morphological data, for example, Buckup (1998) proposed that the Acestrorhynchidae was part of a monophyletic group, the superfamily Erythrinoidea, which included the families Ctenoluciidae, Hepsetidae, Lebiasinidae, and Erythrinidae. At the same time, also based on morphological data, Lucena and Menezes (1998) suggested that the Acestrorhynchidae (*Acestrorhynchus* Eigenmann & Kennedy, 1903) was a sister group of the family Cynodontidae, consisting of the subfamilies Roestinae (*Roestes* Günther, 1864 and *Gilbertolus* Eigenmann & Ogle, 1907) and Cynodontinae (*Cynodon* Spix & Agassiz, 1829, *Hydrolycus* Müller & Troschel, 1844, and *Rhaphiodon* Spix & Agassiz, 1829), and proposed five synapomorphic characters for *Acestrorhynchus*, supporting thus the monophyly of this genus, as proposed by Menezes (1969b) and Menezes and Géry (1983). Nelson et al. (2016) offered a new classification, based on the most recent data adopted for the determination and classification of characiform taxa, in which the Roestinae was placed as a subfamily of the Acestrorhynchidae, with two genera and six species. Two additional acestrorhynchid subfamilies were also identified, the Heterocharacinae, with four genera and six species, and Acestrorhynchinae, with one genus and 14 species.

The genus *Acestrorhynchus* includes piscivorous fishes with an elongated body and snout, conical teeth and robust canines with a characteristic arrangement in the maxilla, together with a number of other diagnostic traits (Menezes 1969a; Menezes and Géry 1983; Toledo-Piza 2007). The species of the genus *Acestrorhynchus* are widespread in South America, where most of its diversity is concentrated in the Amazon and Orinoco river basins, and the rivers of the Guyanas (Nelson et al. 2016).

The *Acestrorhynchus* species can be distinguished on the basis of their coloration patterns and can be allocated to three groups: (i) *Acestrorhynchus lacustris* group (Menezes 1992), characterized by a well-defined dark spot in the humeral region, (ii) *Acestrorhynchus nasutus* group, defined by the presence of two dark, narrow longitudinal stripes, one which extends from the tip of the snout to the base of the caudal fin, and the other, from the posterior margin of the maxilla to the ventral margin of the caudal peduncle (Toledo-Piza 2007), and (iii) the *Acestrorhynchus microlepis* group, diagnosed by the presence of a small dark spot on the anterior region of the body, immediately posterior to the posterior margin of the operculum, at the origin of the lateral line (Toledo-Piza and Menezes 1996; Toledo-Piza 2007). Pretti et al. (2009) analyzed mitochondrial and nuclear sequences from genomes of 11 of the 14 *Acestrorhynchus* recognized species and concluded that the genus should be divided into three groups, although the phylogenetic relationships among these three clades did not correspond with the morphological phylogenies.

The cytogenetic data available for *Acestrorhynchus* indicate a conserved 2n = 50 in all species (Falcão and Bertollo 1985; Martinez et al. 2004; Pastori et al. 2009), although the karyotypes varied both within and among species. For example, in *Acestrorhynchus lacustris* (Lütken, 1875) the karyoytpe is composed of 12m+32sm+4st+2a (Falcão and Bertollo 1985) or 8m+34sm+6st+2a in Martinez et al. (2004). These species have karyotypes with a single pair of NOR-bearing chromosomes, except that of *Acestrohhynchus altus* Menezes, 1969, which has two such pairs (Falcão and Bertollo 1985).

The present study examined the karyotypes and chromosomal characteristics of three Amazonian *Acestrorhynchus* species using both conventional and molecular cytogenetic protocols. Our results were compared with the existing data attempting to better understand the chromosomal differentiation of the genus and the rearrangements involved in this process.

## Material and methods

**Figure 1.**
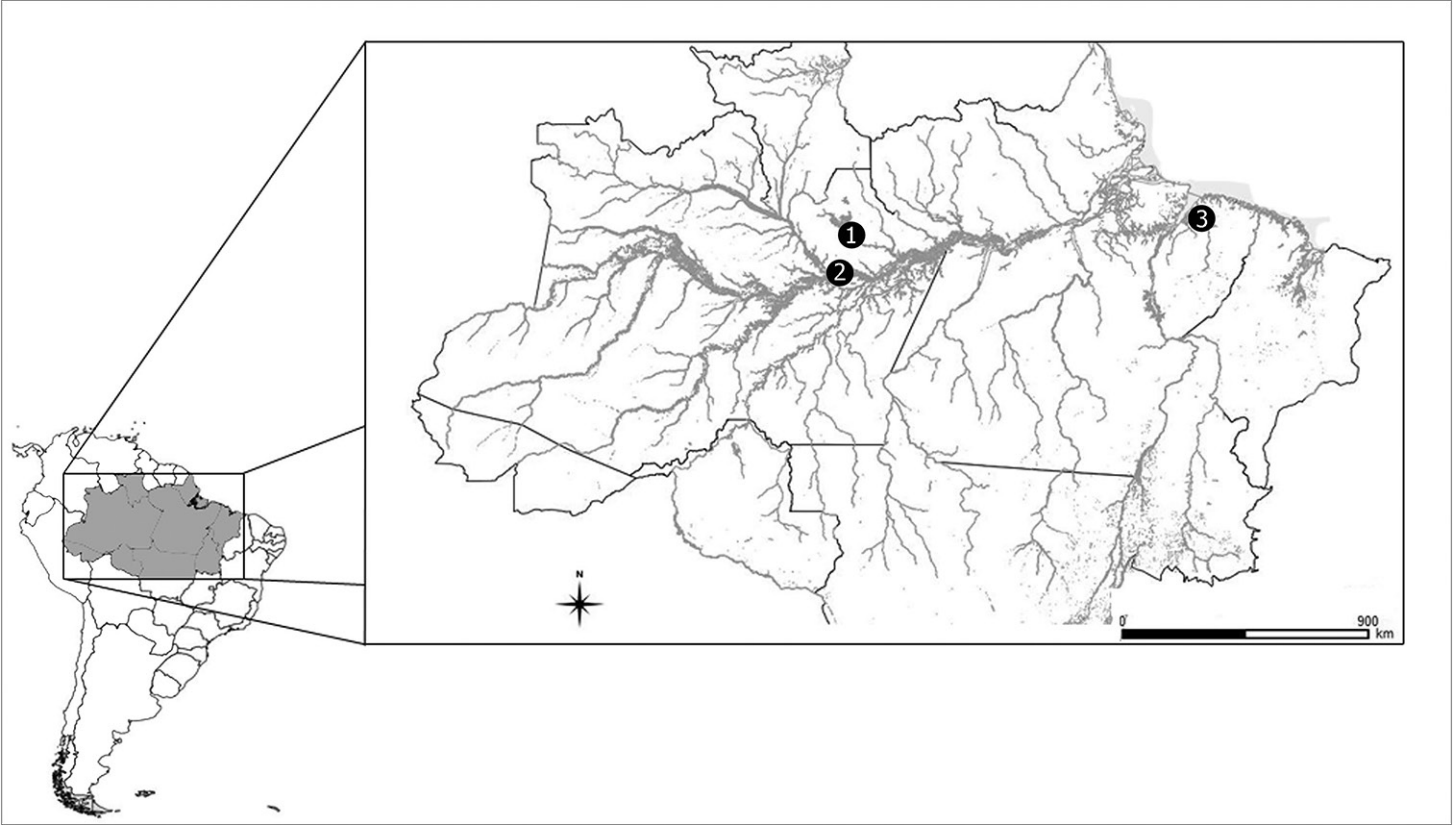
Map of the Brazilian Amazonia region, showing the individual collection localities. **1***Acestrorhynchus falcirostris* – Balbina reservoir on the Uatumã River, Amazonas state **2***Acestrorhynchus falcirostris* and *Acestrorhynchus microlepis* – Catalão Lake, at the confluence of the Negro and Solimões rivers, Amazonas state **3***Acestrorhynchus falcatus*, *Acestrorhycnhus falcirostris*, and Acestrorhynchus prope microlepis – Apeu Stream, basin of the Guamá River, Pará.

The present study analyzed the cytogenetic characteristics of *Acestrorhynchus falcatus* (Block, 1794), *Acestrorhynchus falcirostris* (Cuvier, 1819), and *Acestrorhynchus microlepis*, (Schomburgk, 1841) where the latter species had variation in the chromosome complement of representatives from different collecting localities (Fig. 1, Table 1). The present study followed the ethical standards for zoological research determined by the National Institute of Amazonian Research (INPA) Ethics Committee for the Use of Animals in Research and authorized by protocol number 021/2017. The collection of individuals was authorized by the Brazilian Institute for the Environment and Renewable Natural Resources (IBAMA), through SISBIO license number 28095-1. All the specimens were deposited as vouchers in the INPA Fish Collection (Table 1).

**Table 1.** The *Acestrorhynchus* species included in the present study, collecting localities, and the number of individuals analyzed. ♂ = male; ♀ = female.

Species	Sampling locations	Hydrographic Basin	Coordinates	Number of analized animals	Vouchers
*A. falcatus*	Apeu Stream, Pará, Brazil	Guamá River	1°23'20.4"S, 47°59'07.5"W	8♂ 2♀	INPA 57803
*A. falcirostris*	Catalão Lake, Amazonas, Brazil	Solimões River	3°09'20.4"S, 59°54'47.1"W	1♂ 7♀	INPA 57166
	Balbina UHE, Amazonas, Brazil	Uatumã River	1°55'07.6"S, 59°29'19.7"W	1♂ 2♀	INPA 57167
	Apeu Stream, Pará, Brazil	Guamá River	1°23'20.4"S, 47°59'07.5"W	3♂ 1♀	INPA 57168
*A. microlepis*	Catalão Lake, Amazonas, Brazil	Solimões River	3°09'20.4"S, 59°54'47.1"W	1♂ 2♀	INPA 57599
A. cf. microlepis	Apeu Stream, Pará, Brazil	Guamá River	1°23'20.4"S, 47°59'07.5"W	4♂ 2♀	INPA 57802

### Conventional chromosome banding

The chromosomal preparations were obtained following the protocols of Oliveira et al. (1988) and Gold et al. (1990). The active NORs were detected by silver nitrate impregnation (Ag-NORs), following Howell and Black (1980), while constitutive heterochromatin was detected following Sumner (1972).

### Molecular cytogenetic protocols

The 5S and 18S ribosomal DNA probes were obtained from the genomic DNA of *A. falcirostris*, which was extracted using the Wizard Genomic DNA Purification kit. The rDNA probes were amplified by polymerase chain reaction (PCR), using the primers 18Sf (50-CCG CTG TGG TGA CTC TTG AT-30), and 18Sr (50 - 31 CCG AGGACC TCA CTA AAC CA- 30) (Gross et al. 2010), 5Sa (50-TAC GCC CGA TCT CGT CCG ATC-3’) and 5Sb (5’- CAGGCT GGT ATC GCC GTA AGC-3’) (Martins and Galetti 1999). Telomeric segments were generated using non-templated PCR with primers (TTAGGG)5 and (CCCTAA)5 (Ijdo et al. 1991).

The PCR products were verified in 1.5% agarose gel, and quantified in NanoVue Plus (GE Healthcare). The 18S rDNA gene was marked with digoxigenin-11-dUTP (Dig Nick Translation mix, Roche), while the 5S rDNA gene and telomeric sequences were marked with biotin-14-dATP (Biotin Nick Translation mix, Roche), following the manufacturer´s instructions. The hybridization signals were detected using anti digoxigenin-rhodamine (Roche Applied Science) for the 18S rDNA probe, and streptavidin (Sigma-Aldrich) for the 5S rDNA probes and telomeric sequences. Fluorescence *in situ* hybridization (FISH) was based on the protocol of Pinkel et al. (1986), with a stringency of 77%. The chromosomes were counter-stained with (2 mg/mL) DAPI in a Vectashield (Vector) mounting medium.

### Image analysis and processing

The chromosomes of about 30 metaphases per individual were analyzed and the images were captured using an Olympus BX51 epifluorescence microscope, and processed using Image Pro Plus 4.1 software (Media Cybernetics, Silver Spring, MD, USA). The chromosomes were classified according to Levan et al. (1964).

## Results

**Figure 2.**
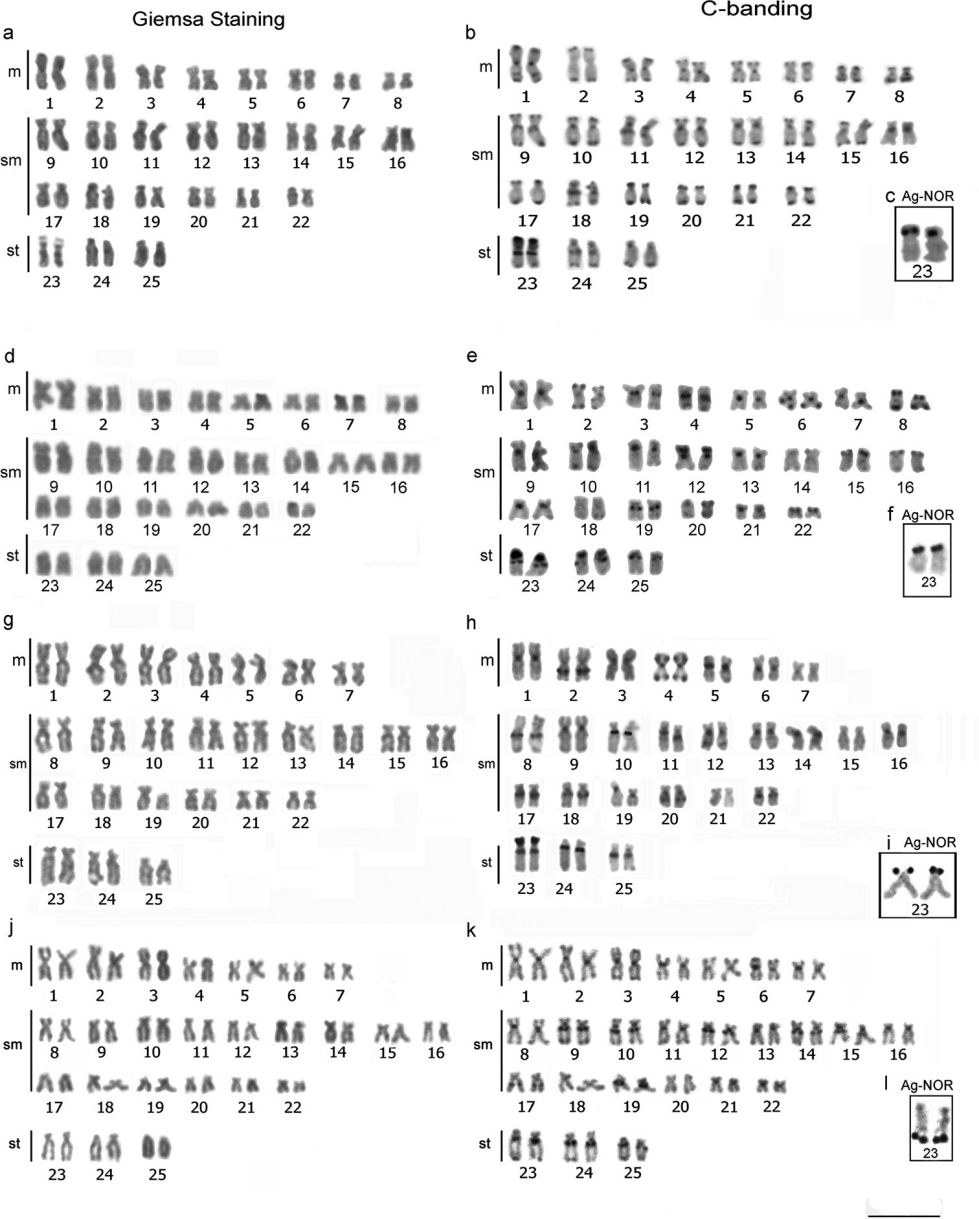
Karyotypes of the species under study arranged from chromsomes stained conventionally with Giemsa, C-banded, and after Ag-NOR impregnation: **a–c***A. falcirostris***d–f***A. falcatus***g–i**A. prope microlepis**j–l***A. microlepis*. Scale bar: 10 µm.

All *Acestrorhynchus falcatus*, *A. falcirostris*, *A. microlepis*, and A. prope microlepis individuals possessed invariably 2n = 50 and a fundamental number (FN) 100. Their karyotypes were very similar to each other and composed of 16m+28sm+6st in *A. falcirostris* and *A. falcatus*, while 14m+30sm+6st in *A. microlepis* and A. prope microlepis (Fig. 2a, d, g, j).

The NORs were located in a distal position on the *p* arms of pair No. 23 in all the species, except for *A. microlepis*, in which the NORs were located on the *q* arms of pair No. 23 (Fig. 2c, f, i, l).

**Figure 3.**
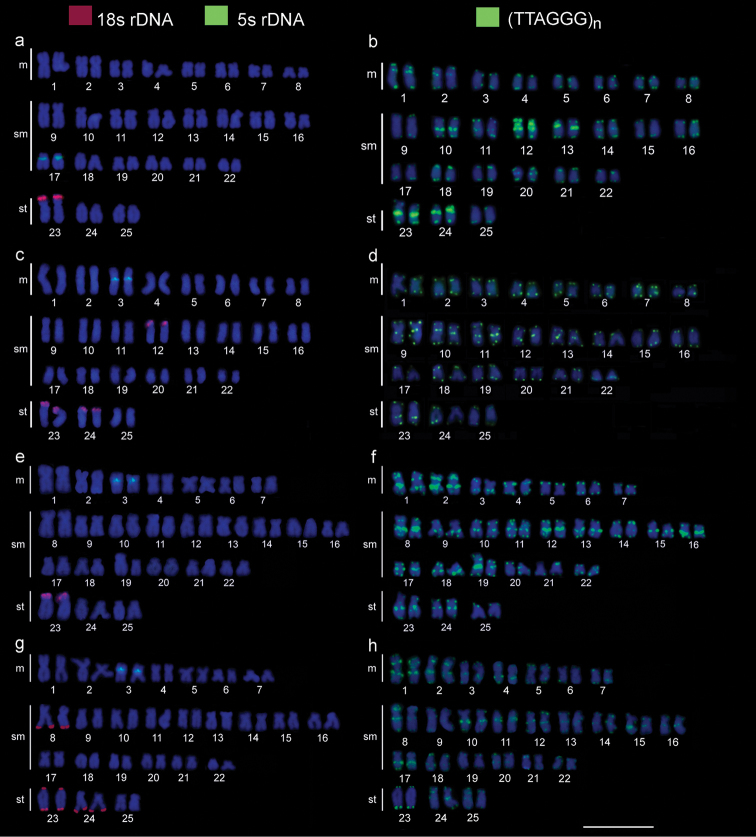
Karyotypes of the species under study, arranged from chromosomes showing “double” FISH with 18S rDNA (red) and 5S (green) probes **a, c, g** and FISH with (TTAAGG)n probe, in green **b, d, f, h***A. falcirostris* (**a, b**), *A. falcatus* (**c, d**), A. prope microlepis (**e, f**), *A. microlepis* (**g, h**). Scale bar: 10 µm.

The positive 18S rDNA sites corresponded to the NOR signals in *A. falcirostris* and A. prope microlepis, at pair No. 23 (Fig. 3a, c, e, g), whereas in *A. falcatus* and *A. microlepis*, the 18S rDNA sites were observed at two chromosome pairs in addition to the single NOR-bearing pair. In *A. falcatus*, these additional 18S rDNA loci resided on the *p* arms of pairs Nos. 12 and 24 (Fig. 3c), while in *A. microlepis* they mapped to the *q* arms of pairs Nos. 8 and 24 (Fig. 3g).

The blocks of constitutive heterochromatin were distributed in centromeric and telomeric regions in karyotypes of all species, though with unique features found in each species, as follows:

*A. falcatus*: heterochromatin in centromeric and telomeric blocks in pairs Nos. 2, 4, 6, 8, 10, 15, 16, 20, and 22, and in centromeric blocks only in pairs Nos. 1, 3, 5, 7, 9, 11, 13, 14, 17, 19, and 21, while pairs Nos. 12, 23, 24, and 25 have entirely heterochromatic p arms, and pair No. 18 had no clear heterochromatic signal (Fig. 2e).

*A. falcirostris*: heterochromatin in centromeric and telomeric blocks in pairs No. 1, 3, 4, 5, 7, 8, 9, 10, 12, 14, 15, and 18, in telomeric blocks only in pairs Nos. 2, 6, 17, 19, 20, 21, and 22 and in pericentromeric blocks only in pairs Nos. 11, 16, 23, and 24. Pairs Nos. 13 and 25 have centromeric blocks and terminal blocks on the q arms. In pair No. 23, a differential accumulation of heterochromatin was observed in the *p* arms, with blocks adjacent to the NOR (Fig. 2b).

A. prope microlepis: heterochromatin in centromeric and pericentromeric regions. Pair No. 4 also had telomeric signals, while in pairs Nos. 2, 8, 17, 18, 20, 23, 24, and 25, there is a block in a more interstitial position. Pair No. 19 displayed size heteromorphism of a heterochromatin block, observed after both Giemsa staining and C-banding (Fig. 2h).

*A. microlepis*: heterochromatin found primarily in centromeric regions, with some proximal signals, but in a pattern distinct from that observed in A. prope microlepis, in terms of the location and position of the heterochromatin on some chromosome pairs (Fig. 2k).

The mapping of the 5S rDNA gene revealed a pericentromeric signal in only one pair in each species (pair No. 17 in *A. falcirostris*, pair No. 3 in *A. falcatus*, A. microlepis and A. prope microlepis) (Fig. 3a, c, e, g).

**Figure 4.**
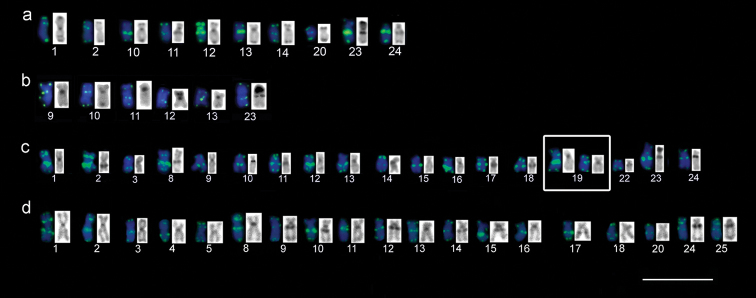
Chromosomal pairs with ITSs in comparison with C-positive (C-banding) heterochromatin **a***A. falcirostris***b***A. falcatus***c**A. prope microlepis**d***A. microlepis*. The pairs are arranged irrespective of the type of chromosome. Scale bar: 10 µm.

Telomeric sequences were detected in the terminal regions of all chromosomes, as expected, but also with additional interstitial telomeric sequences (ITSs) in a number of chromosome pairs in all species under study, displaying species-specific patterns in terms of their localization (Fig. 3b, d, f, h). In *A. falcirostris*, the ITSs were located in 10 chromosome pairs, with an accumulation of these sequences in pairs Nos. 12, 13, 23, and 24 (Fig. 4a). In *A. falcatus*, the ITSs were found in six chromosome pairs (Fig. 4b), while in A. prope microlepis, they were present in 18 pairs, displaying varied signal intensities; and in pair No. 19, the ITSs varied in size between the homologs (Fig. 4c). In *A. microlepis*, ITSs were present in 19 pairs (Fig. 4d).

## Discussion

All species analyzed in the present study have invariably 2n = 50 chromosomes, with no morphologically distinguishable sex chromosomes. There is a considerable variation, however, in the karyotype structures and the FN values (Falcão and Bertollo 1985; Martinez et al. 2004; Pastori et al. 2009; present study). One of the main differences between the present study and the formerly published data is the absence of acrocentric chromosomes in the karyotypes of species analyzed here (Table 2). However, these analyzed species encompass all three morphological groups (based on coloration patterns) defined by Menezes (1969a), Menezes and Géry (1983), and Toledo-Piza (2007), i.e., the *Acestrorhynchus lacustris* group (*A. falcatus*), *A. nasutus* group (*A. falcirostris*), and *A. microlepis* group (*A. microlepis* and A. prope microlepis). Considering these morphological groups for the genus *Acestrorhynchus*, no group-level cytogenetic marker was found (Table 2).

**Table 2.** Cytogenetic data available for the representatives of the genus *Acestrorynchus*. (2n = diploid chromosome number, FN = Fundamental Number, NOR = Nucleolus Organizer Region, ITS = Interstitial Telomeric Sequence, m = metacentric, sm = submetacentric, st = subtelocentric, a = acrocentric chromosomes p = short arm, q = long arm).

Groups	Species	2n	FN	NOR	Karyotype formulae	N° and location 18S rDNA	N° and location 5S rDNA	N° of pairs ITS	References
*** lacustris ***	*A. altus*	50	94	2 pairs	8m+22sm+14st+6a	-	-	-	Falcão and Bertollo [1985]
	*A. falcatus*	50	100	1 pair	16m+28sm+6st	3 pairs; (p)	1 pair (3); pericentromeric	6 pairs	Present study
	*A. lacustris*	50	98	1 pair	12m+32sm+4st+2a	-	-	-	Falcão and Bertollo [1985]
	*A. lacustris*	50	98	-	8m+34sm+6st+2a	-	-	-	Martinez et al. [2004]
	*A. pantaneiro*	50	86	1pair	36 m-sm+14st-a	-	-	-	Pastori et al. [2009]
*** microlepis ***	A. cf. microlepis	50	100	1pair	14m+30sm+6st	1 pair; (p)	1 pair (3); pericentromeric	18 pairs	Present study
	*A. microlepis*	50	100	1pair	14m+30sm+6st	2 pairs; (q) and 1 pair bitelomeric	1 pair (3); pericentromeric	19 pairs	Present study
*** nasutus ***	*A. falcirostris*	50	100	1 pair	16m+28sm+6st	1 pair; (p)	1 pair (17); pericentromeric	10 pairs	Present study

Based on the analysis of morphological characters, Lucena and Menezes (1998), Toledo-Piza (2007) and Mirande (2010) reached the same conclusion that the family Acestrorhynchidae is a sister group of the Cynodontidae, which has a known 2n = 54 (Arai 2011). This would suggest that the ancestral karyotype of *Acestrorhynchus* would have had 54 biarmed chromosomes, which evolved likely through fusions, reducing thus the 2n; and inversions, or reciprocal/nonreciprocal translocations, or centromere repositioning, or heterochromatin loss/addition resulting in the maintenance of the complement of biarmed chromosomes, but with distinctly different karyotypes.

A similar scenario is found in the Erythrinoidea (Ctenoluciidae, Hepsetidae, Lebiasinidae and Erythrinidae), a fish groups that are also closely-related to the Acestrorhynchidae (Ortí and Meyer 1997; Buckup 1998). Except for the Hepsetidae, which has 2n = 58 (Carvalho et al. 2017), there has been a reduction in the 2n. In the Erythrinidae, for example, the 2n ranges from 40 to 50 (Oliveira et al. 2015), while most representatives of Lebiasinidae possess 2n = 40 (Moraes et al. 2017), and those of Ctenoluciidae have 2n = 36 (Sousa e Souza et al. 2017).

The comparison of the different markers provides valuable insights into the chromosomal differentiation of *Acestrorhynchus*. In karyotypes of all species, the blocks of constitutive heterochromatin are located primarily in centromeric or telomeric regions, although large heterochromatic blocks are associated with the NORs, as seen as in most species of teleost fish of different families of different orders such as Anguilliformes, Siluriformes, Characiformes, among others (Gornung 2013; Blanco et al. 2014; Salvadori et al. 2018). The NOR phenotype was simple, i.e. one pair of NOR-bearing chromosomes, as observed also in Ctenoluciidae (Sousa e Souza et al. 2017), Cynodontidae (Pastori et al. 2009), and some Erythrinidae species (Bertollo 2007), although multiple NORs are also found in the Lebiasinidae (Moraes et al. 2017). Two species, *A. falcatus* and *A. microlepis*, have multiple 18S rDNA signals, but the Ag-NOR was simple. On the other hand, Falcão and Bertollo (1985) observed multiple Ag-NORs (two pairs) in karyotype of *A. altus* from the Miranda River, Mato Grosso do Sul. As terminal chromosomes regions may be more vulnerable to the transfer of genetic material due to their proximity in the nucleus, (Schweizer and Loidl 1987), the dispersal of the 18S rDNA sequences in *A. falcatus* and *A. microlepis* may have been facilitated by their proximity to the telomere or by ectopic recombination in meiosis (Pedrosa-Harand et al. 2006; Cazaux et al. 2011; Evtushenko et al. 2016).

The telomeric sequence was a particularly valuable cytogenetic marker, with a species-specific configuration in the four studied taxa, due to the large number of ITSs distributed in different pairs. In fishes, as in other vertebrates, the pericentromeric ITSs are found within or adjacent to the constitutive heterochromatin (Milhomem et al. 2008; Cioffi et al. 2010; Scacchetti et al. 2011; Rosa et al. 2012; Ocalewicz 2013). The ITSs can be classified in six types: heterochromatic (het-ITSs), short (s-ITSs), large ITSs in restricted euchromatic regions (Restricted eu-ITSs), long subtelomeric, fusion, and pericentromeric ones (Lin and Yan 2008; Ruiz-Herrera et al. 2008; Schmid and Steinlein 2016).

In *Acestrorhynchus* species all six ITS types have been observed. Larger sequences were observed in association with the blocks of constitutive heterochromatin in some chromosome pairs as revealed by the C-banding, although a number of the observed ITSs were not associated in any way with the heterochromatin (Fig. 4). It is possible that the het-ITS arose as short sequences through processes such as repair mechanisms (Nergadze et al. 2004, 2007), fusion (Slijepcevic 1998; Bolzán and Bianchi 2006), transposition (Bouffler et al. 1993; Nergadze et al. 2007) or in association with satellite DNA as seen in a species of the family Sparidae (Perciformes) (Garrido-Ramos et al. 1998). These sequences would have increased in length through duplication, in specific independent events in each species, which would then have become integrated with the heterochromatin and become detectable by FISH (Nergadze et al. 2004, 2007; Bolzán and Bianchi 2006).

Other types of ITS, not associated with the heterochromatin would have arisen through terminal translocations, the insertion of telomeric repetitions during the repair of breaks in double-strand DNA, or by the duplication or transposition of genes (Lin and Yan 2008; Ruiz-Herrera et al. 2008; Bolzán 2012). Ruiz-Herrera et al. (2008) concluded that the occurrence of het-ITS is related to the expression of the genes of a specific cellular lineage through epigenetic modifications. No specific function is known in the case of the ITSs that are unrelated to the heterochromatin, although this does not impede their inclusion in the analysis of the evolutionary history of closely-related species. As the chromosomal evolution of *Acestrorhynchus* appears to have been based on a reduction of the number of chromosomes, some of the ITSs may actually be remains of specific rearrangements, although a definitive understanding of this process will require more detailed data from a larger number of species.

In the specific case of *A. microlepis*, remarkable differences were found between the individuals collected at the two localities (Catalão Lake and Apeu Stream, respectively), both in the location of the NORs and the number and location of the 18S rDNA sites. Thus the individuals of Pará (Apeu) were provisionally named A. prope microlepis. These chromosomal differences may reflect the presence of an unnamed species, that is, a past speciation event, which would have been caused by the geographic distance between the two populations. This distance would have minimized gene flow, isolating the populations, and permitting the fixation of specific rearrangements. A probable rearrangement was a pericentric inversion involving the NOR carrier pair, since NOR in three species was on the short arm and *A. microlepis* was on the same pair, but located on the long arm.

Another possible type of arrangement is the translocation of major ribosomal 18S sites, which were present in four other sites, in addition to the NORs. This movement may have been facilitated by transposable elements (TEs) associated with the heterochromatin, which has great potential to cause chromosomal rearrangements, as well as through ectopic recombination that can generate intrachromosomal recombination between copies of the same family of transposable elements, arranged in opposite positions (Kidwell 2002; Grewal and Jia 2007; Skipper 2007; Raskina et al. 2008; Delprat et al. 2009; Cioffi et al. 2010; Evtushenko et al. 2016). The genomes of *A. microlepis* and A. prope microlepis differed also in terms of their ITSs, given not only that the ITSs were present in 19 chromosome pairs in one species, and in 18 pairs in the other one, but also the fact that these chromosomes were different, as well as the polymorphism between the homologs of pair 19 in A. prope microlepis. In this case, there was a larger ITS in one of the homologs, indicating the translocation of a telomeric sequence to this chromosome (Ruiz-Herrera et al. 2008; Bolzán 2017) and its duplication. An ITS may indicate the presence of chromosomal rearrangements during the evolutionary process, leading to the differentiation of the karyotypes of different species, as observed in several fish families (Meyne et al. 1989; Mota-Velasco et al. 2010; Cioffi and Bertollo 2012; Ocalewicz 2013; Sousa e Souza et al. 2017).

López-Fernández and Winemiller (2003) found subtle differences in the pigmentation and body shape of individuals identified as *A. microlepis*, but concluded that this variability was not sufficient to differentiate species. Furthermore, these authors concluded that *A. apurensis* Toledo-Pizza & Menezes, 1996, described from the Orinoco River in Venezuela was in fact a junior synonym of *A. microlepis*, which occurs in the Negro and Branco rivers in northern Brazil, and in the river basins of the Guyanas. However, the results of the present study indicates that the *A. microlepis* and A. prope microlepis individuals, while morphologically very similar, have karyotypes with significantly different locations of their NORs and 18S rDNA sites, C-banding patterns, and the pattern of ITSs, including the polymorphism of the homologs of pair No. 19 in A. prope microlepis.

Cioffi et al. (2018) highlighted the importance of cytogenetic markers for the identification of morphologically similar and/or identical fish groups, as in the case of the *Hoplias malabaricus* (Bloch, 1794), which had seven distinct karyomorphs, including some found in sympatry, supporting the need for a taxonomic review of this group. Cytotaxonomic markers are also useful for the differentiation of species that are often misidentified due to the morphological similarities, as observed in the pike-characins *Boulengerella lucius* (Cuvier, 1816) and *B. maculata* (Valenciennes, 1850), karyotypes of which have distinct patterns of differentiation of the 5S rDNA sequences (Sousa e Souza et al. 2017). Overall, then, certain specific features of the karyotype microstructures of the species analyzed here were found to be diagnostic characters for the diagnosing the diversity of the genus *Acestrorhynchus*.
